# An extensive re-evaluation of evidence and analyses of the Randomised Badger Culling Trial (RBCT) I: Within proactive culling areas

**DOI:** 10.1098/rsos.240385

**Published:** 2024-08-21

**Authors:** Cathal L. Mills, Rosie Woodroffe, Christl A. Donnelly

**Affiliations:** ^1^ Department of Statistics, University of Oxford, Oxford, UK; ^2^ Pandemic Sciences Institute, University of Oxford, Oxford, UK; ^3^ Institute of Zoology, Zoological Society of London, London, UK

**Keywords:** badgers, epidemiology, tuberculosis, culling, cattle

## Abstract

Here, in the first of two investigations, we evaluate and extend the analyses of the Randomised Badger Culling Trial (RBCT) to estimate the effectiveness of proactive badger culling for reducing incidence of tuberculosis (TB) in cattle within culling areas. Using previously reviewed, publicly available data, alongside frequentist and Bayesian approaches, we re-estimate culling effects for confirmed incidence of herd breakdowns (TB incidents in cattle) within proactive culling areas. We appraise the varying assumptions and statistical structures of individual models to determine model appropriateness. Our re-evaluation of frequentist models provides results consistent with peer-reviewed analyses of RBCT data, due to the consistency of beneficial effects across three analysis periods. Furthermore, well-fitting Bayesian models with weakly informative prior distribution assumptions produce high probabilities (91.2%–99.5%) of beneficial effects of proactive culling on confirmed herd breakdowns within culling areas in the period from the initial culls (between 1998 and 2002) until 2005. Similarly high probabilities of beneficial effects were observed post-trial (from 1 year after last culls until March 2013). Thus, irrespective of statistical approach or study period, we estimate substantial beneficial effects of proactive culling within culling areas, consistent with separate, existing, peer-reviewed analyses of the RBCT data.

## 1. Introduction

### 1.1. Bovine tuberculosis (TB) in Great Britain

Bovine tuberculosis (TB) is an infectious zoonotic disease caused by *Mycobacterium bovis*. European badgers (*Meles meles*) have been identified as wildlife hosts for *M. bovis*. Due to the ecological, public health and economic consequences of bovine TB, badger culling strategies were implemented in attempts to curb transmission of *M. bovis* and thus, reduce incidence of TB in cattle across Great Britain.

In England, the Randomised Badger Culling Trial (RBCT) was a large-scale randomised field trial conducted in 30 trial areas of similar size (approximately 100 ${\text{km}}^{2}$) with high incidence of herd breakdowns (i.e. cattle TB incidents). The trial was designed and overseen by a group of independent scientists, collectively known as the Independent Science Group (ISG) on Cattle TB [1]. Initial culling activity of the RBCT began in 1998 and culls were performed approximately annually (excluding the interruptions caused by the foot-and-mouth disease epidemic in 2001). The final culling activity of the RBCT took place in 2005 [2].

The RBCT study design, established pre-trial in 1998 [1], comprised unculled comparison (or survey-only) areas which were compared to areas under two badger culling (or treatment) strategies; proactive culling and reactive culling. Proactive culling involved removing as large a proportion as possible of badgers initially resident in the trial area at the beginning of the trial, and carrying out regular follow-up culling activity to achieve sustained population suppression. Alternatively, (localized) reactive culling only took place in the event of a cattle herd experiencing a confirmed herd breakdown. The objective of the RBCT was to appraise the effectiveness of the two badger culling strategies to reduce TB incidence in cattle herds within a pre-specified trial duration period of a total of 50 triplet-years [3,4]. The 30 trial areas were collectively grouped into sets of three called triplets and there was randomization of treatments (a procedure which was independently witnessed) within each triplet. Trial areas were separated by at least 3 km [5]. No licensed badger culling occurred outside the trial areas. Aside from the aforementioned statistical considerations, the RBCT study design featured further ecological and animal welfare considerations, such as the avoidance of badger trapping between February and April (inclusive) of each year [6].

The control of bovine TB remains a key policy challenge in Great Britain. Each year, the Department for Environment, Food and Rural Affairs (Defra) publishes minimum and maximum badger cull numbers in areas of England with general cull licences [7]. The minimum badger cull numbers are proposed as a means to achieve a 70% reduction of the badger population, a target informed by analyses of the RBCT [8]. Natural England, the body responsible for issuing new culling licences, also accepts applications for supplementary cull licences (designed for areas which have already undergone 4 years of intensive culling) which are subject to public consultations [9]. In Wales, widespread badger culling does not take place and the Bovine TB Eradication Programme has outlined the government’s ambition to prohibit badger culling and instead promote a badger vaccination policy [10]. There has been a general long-term trend in Wales of declining herd incidents over the past decade and 94.6% of herds were estimated to be TB-free as of Q3 2023, yet recent trends include 1.7% and 6.4% increases to the reported number of animals slaughtered and new herd incidents between October 2021 to September 2022 and October 2022 to September 2023 [11].

### 1.2. Analyses of within-trial areas of the Randomised Badger Culling Trial (RBCT)

The statistical analyses of the RBCT were pre-defined (before the incidence data were collected) and also independently audited by a statistical auditor [1,3,4]. The response variables of interest for the analyses were the observed: (i) total number of herd breakdowns (inclusive of confirmed and unconfirmed) and (ii) number of confirmed herd breakdowns [12]. Confirmed herd breakdowns were herd incidents which involved evidence of TB exposure in at least one cattle herd member, and either lesions characteristic of TB were subsequently identified at post-mortem or the *M. bovis* organism was cultured. Otherwise, breakdowns were classified as unconfirmed. The results of an interim analysis in 2003 led to the suspension of the reactive badger culling strategy due to a demonstrated increased risk of confirmed cattle herd breakdowns [5]. Subsequent ecological studies investigated the estimated increased TB risk induced by reactive culling and observed differences to badger ecology and behaviour, as well as *M. bovis* epidemiology in badgers. Increased ranges, increased mixing and hence greater *M. bovis* transmission and prevalence within badger populations were estimated, as well as increases to the number of cattle herds potentially in contact with each infected badger [13–16].

The findings for proactive culling, of beneficial effects within culling areas, were consistent across the 10 trial area pairs [12,17], while the beneficial effect of proactive culling increased with distance from the boundary and with successive annual culls [2]. Post-trial analyses, following the cessation of RBCT culling, estimated that benefits inside cull areas had declined over time and by 2010, were not detectable [18,19]. Subsequently, results were updated on the basis of the most recent six month interval of data, which estimated the subsequent beneficial effects within trial areas. By 2013, a complementary analysis of a longer post-trial period estimated that post-trial incidence of confirmed herd breakdowns within the proactive culling areas was 25.9% lower compared with survey-only areas, while also being 6.8% lower compared with areas up to 2 km outside survey-only areas. The estimated beneficial effects of culling within inner trial areas persisted to the last six month period analysed (over 78 months) [20].

Recently, a separate preprint manuscript [21] questioned both the validity of the methodology and conclusions from several peer-reviewed, audited analyses of RBCT data [5,12,18,19,22]. In particular, the authors reported that the statistical modelling approach, the log-linear Poisson Generalized Linear Model (GLM), was sub-optimal and likely overfitted the data. Furthermore, the review concluded that alternative modelling approaches, such as a quasi-Poisson model or a model with offset variable specification, did not imply the same beneficial effects of culling on incidence of TB in cattle in proactive culling trial areas. There have also been analyses of the industry-led culling undertaken since 2013 as TB control policy, rather than as part of a controlled field trial. Three analyses found reduced TB incidence rates in the culled areas [23–25], although another analysis found no such effect [26]. As industry-led culling was not randomised to areas and it has been so widespread in areas of England with high TB herd incidence (limiting areas available for comparison), in this context, fully robust causal analyses are not possible due to potentially confounding factors. Here, our analysis is strictly focused on the evidence from the RBCT, a trial which was subjected to the aforementioned independently audited study design including culling and survey-only areas.

### 1.3. Objectives of the current analysis

The primary objective of the current analysis is to comprehensively re-assess the available evidence from the RBCT regarding the effects of proactive culling on incidence of herd breakdowns in cattle populations within proactively culled areas. Our in-depth re-evaluation explores modelling approaches beyond those taken previously by the ISG, by other subsequent analyses [27,28], and by a recent, separate preprint manuscript [21], and we subject each approach to the same rigorous model checking and model comparison. We aim to comprehensively examine the statistical issues of each modelling approach and subsequently, deliver our evidence-based findings about the beneficial effects within proactively culled areas.

Using previously published data, our analysis considers two during-trial periods and one post-trial period. We further consider a range of Bayesian statistical approaches to modelling. Our extension to a wide array of statistical techniques and study periods allows us to make robust conclusions regarding the effects of proactive badger culling which are informed by consistent scientific evidence from trial data, irrespective of which approach to statistical inference is taken. Separately, our complementary analysis examines the effects of proactive culling in neighbouring areas up to 2 km outside the culling areas across the same three periods (two during-trial periods and one post-trial period) [29].

## 2. Material and methods

### 2.1. Data

Our studied during-trial periods for our analysis of the RBCT data (previously published in [22]) cover: (i) from each triplet’s *initial* proactive cull until 4 September 2005 and (ii) from each triplet’s first *follow-up* proactive cull until 4 September 2005. 4 September 2005 represents the last during-trial date for which trial data were available for the analysis by [22], while a recent preprint manuscript also used data from initial cull until 4 September 2005 [21]. The during-trial data amount to respective totals of: (i) 46.6 triplet years since the initial cull and (ii) 34.1 triplet years since the first follow-up cull. The post-trial period consists of 66.6 triplet years, from 1 year after the last cull until 28 March 2013. In each analysis, our primary variable of interest is the number of *confirmed* herd breakdowns. We present results for the total number of breakdowns, inclusive of unconfirmed breakdowns (which represent positive tests for *M. bovis* in live cattle which were not confirmed at post-mortem) in electronic supplementary material, §6.2. Across each dataset, the following information is also available for each trial area: herd-years-at-risk, historical 1 year incidence of cattle TB, historical 3 year incidence of cattle TB and baseline number of herds. Thus, there are a range of potential covariates (or offsets) for a model of herd breakdowns.

### 2.2. Statistical approach

In general, due to the statistical nature of our response (observed herd breakdowns represent count data), we note the potential for the phenomenon of overdispersion which commonly arises in count data due to unaccounted-for heterogeneity in the response. The variability being greater than the mean is known as overdispersion. In the case of a Poisson regression model, the dispersion parameter is effectively set to 1. Overdispersion was initially addressed in the various peer-reviewed analyses of the RBCT [2,5,22]. The ISG noted that the observed variance in herd breakdowns was greater than the theoretical variance predicted by the proposed Poisson regression model. Hence, the ISG reported parameter estimates and confidence intervals (CIs) with an adjustment for overdispersion. Specifically, the ISG estimated the overdispersion factor as the square-root of the deviance divided by the degrees of freedom, and CIs were subsequently adjusted for the extra-Poisson overdispersion when the estimated overdispersion factor was greater than 1 [12]. Another possible option for a Poisson regression model is the usage of an offset variable which enables modelling the count variable (here confirmed herd breakdowns) as a rate, and the usage of an offset variable means that the corresponding regression coefficient is constrained to be 1. In a Poisson count regression, the offset variable can be the logarithm of measurement time. A key limitation of using an offset variable is that there is a strong and potentially restrictive underlying statistical assumption with important scientific implications. In particular, when using an offset, one assumes that doubling the measurement time, on average, leads to the doubling of the count outcome. Alternatively, assuming an offset variable not be supported by evidence (i.e. the number of events may increase non-proportionally with the population at risk) [30], one could use an unconstrained regression coefficient and hence, instead of assuming the slope for the variable is exactly 1, the slope parameter is estimated.

The statistical analyses for the RBCT data, led by the ISG and regularly reviewed by an independent auditor, involved the usage of a log-linear Poisson regression which modelled the observed herd breakdowns [1–5,22,31]. The Poisson regression model examined treatment/culling effects and made adjustments for factors such as individual triplet effect, the log of the number of baseline herds-at-risk, and the log of the historic 3-year incidence of herd breakdowns. Alternative model formulations were appraised, alongside the sensitivity of inferences. Other formulations included covariate interactions with culling effects, different time periods and adjustments for log of the baseline total cattle numbers and log of the total number of tests conducted. Results obtained were similar across each of the different modelling environments. In the current re-evaluation, the primary variable of interest is the number of confirmed herd breakdowns within proactive culling areas in the respective studied periods (detailed in §2.1). Our models estimate the effects of proactive culling by drawing comparison between the confirmed herd breakdowns in proactive culling (treatment) areas and the confirmed herd breakdowns in the corresponding/paired unculled (survey-only) areas. The models considered in the current analysis provide the most extensive assessment of the impacts of proactive culling from the RBCT, as the models adopt differing parametric families (e.g. Poisson versus quasi-Poisson), specifications (e.g. covariate versus offset forms), and approaches to statistical inference (both frequentism and Bayesianism) across three studied periods (two during-trial period and one post-trial period). Indeed, by also adopting a Bayesian perspective to modelling in each studied period (to make probabilistic statements about the effects of proactive culling), we have extended beyond the frequentist approaches taken by the ISG. Note that our separate, complementary analysis explores the number of confirmed herd breakdowns in neighbouring areas immediately outside proactive culling areas [29].

### 2.3. Model appraisal and diagnostics

Across the two paradigms of frequentism and Bayesianism (further detailed in electronic supplementary materials, 3 and 4), our model appraisal involves an assessment of the validity of underlying scientific assumptions which enable statistical inference (see electronic supplementary material, 5). It is important to assess the inherent implications of each model structure. For example, a quasi-Poisson model structure means that standard errors and CIs are allowed to be inflated and widened. Thus, we assess whether such model structures are scientifically plausible and whether individual model fits (relative to other proposed models) across the study periods support the proposed structures. We employ additional model diagnostics to identify poorly specified models (either in terms of structure or the moderators/covariates included). Our priority here is not exclusively predictive, as the modelling objective is to understand the effects of the intervention of proactive culling (the treatment) on cattle TB. In recognition of the fact that any statistical model represents a simplification of underlying scientific processes, we retain and present all models alongside commentary of the models’ strengths and weaknesses. We advocate caution when interpreting values directly from summary tables as assumptions of the proposed models vary, alongside their statistical and scientific appropriateness. Therefore, we outline the statistical structure, scientific context and moderators included in the model when appraising the direct treatment (culling) effect.

## 3. Results

We present results across frequentist and Bayesian statistical approaches, for data from initial cull until 4 September 2005 (the last date for which trial data were available for Donnelly *et al*. [22]), and for data from the post-trial period (1 year after last culls until March 2013). Results for incidence data from first follow-up cull until 4 September 2005 are included in electronic supplementary material, §§6.1.1 and 7.2.

### 3.1. Frequentist approaches to modelling

#### 3.1.1. From initial cull until 4 September 2005

The following frequentist models were fitted to confirmed herd breakdowns within proactive culling areas and survey-only areas from initial culls of the RBCT until 4 September 2005. The incidence data analysed here are the same previously published, publicly available data from Donnelly *et al*. [22]. Tables 1a,b contain a summary of our key results, while sample model diagnostics and checks are displayed in electronic supplementary material, §6.1.

**Table 1a.** For confirmed herd breakdowns from initial cull until 4 September 2005 within proactive culling areas of the RBCT, we present a range of frequentist models. Various Poisson GLMs were examined across analyses of the RBCT led by the ISG and a final Poisson GLM was fitted to incidence data for the period from initial cull until 4 September 2005 in [22]. The model was independently audited by a statistical auditor who certified the accuracy of the findings and the associated interpretations [32,33]. In a separate preprint manuscript by Torgerson *et al*. [21], variants of the model were proposed, each of which are denoted by an asterisk (*) below. BIC and AICc denote the Bayesian Information Criterion and small-sample corrected Akaike Information Criterion, respectively. Lower values of each information criterion are better but note that it is not appropriate to compare the information criteria of the normal linear model (which assumes a continuous response and assumes normality of errors in a different model fitting method) with the Poisson-based models.

model	structure	estimated effect Of culling (95% CI)	BIC	AICc	comments
1	original Poisson GLM (from [22])	−18.7% (−29.4%, −6.2%)	155.2	203.0	results attained are identical to those previously published in existing analyses [22] quantile deviation is detected in the simulated residual versus predicted plot. *p*‐value 0.001) across the Quantile–Quantile(QQ)-Plot-based diagnostic checks of simulated residuals, non-significant results are attained for uniformity and deviations from uniformity but the test of overdispersion or underdispersion yields a significant result (*p*‐value < 0.001) a visual posterior predictive check indicates that the model’s posterior predictive distribution resembles the observed data. no influential observations are detected via leverage plots or Cook’s Distance
2	quasi-Poisson GLM *	−13.6% (−31.3%, 8.6%)	NA	NA	due to the quasi-Poisson model structure, we cannot diagnose model fit using simulated residuals nor readily compare to other models. In particular, the quasi-likelihood prevents testing for over/underdispersion an influential observation (Triplet H-Survey-Only) is identified (electronic supplementary material, figure S1) due to Cook’s Distance > 1, high leverage, and a large studentized residual. Indeed, the Cook’s Distance value for the observation is over four times as large as any other distance value the quasi-Poisson’s primary effect is unveiled as it yields a more flexible fit and substantially larger standard errors for the coefficient estimates. Such increased flexibility may not always be desirable, especially for generalization as the likelihood is not assumed to be any defined probability distribution, information criteria, posterior predictive checking and other useful statistical tests are not applicable to provide rigorous comparisons with other model fits nor to validate the model
3	original GLM (from [22]) in generalized Poisson form	−18.7% (−24.6%, −12.3%)	147.1	217.2	the QQ-plot for simulated residuals (electronic supplementary material, figure S3) indicates no significant over- or underdispersion, no significant outliers, and we fail to reject the Kolmogorov–Smirnov (KS) test for uniformity of the scaled residuals the simulated residual versus predicted plot (electronic supplementary material, figure S3) indicates some deviation, with a greater number of residuals in the tail of the distribution (around 0) than would be expected. However, consistent with the capabilities of the DHARMa package, with a low number of data points (here, we have only 20 observations), implied deviation can be a consequence of statistical chance the posterior predictive check (Figure) indicates close correspondence with the confirmed incidence data the finding of a significant, beneficial effect of culling on incidence is within the context of the model being the frequentist GLM with the lowest BIC across the period
4	generalized Poisson (without any culling effect) *	NA	162.0	160.4	the QQ-plot indicates no significant overdispersion or underdispersion, no significant outliers, and we fail to reject the KS test for uniformity of the scaled residuals no deviations are detected in the residual versus predicted plot however, the posterior predictive check indicates potential model misfit as the model’s predicted lines do not closely resemble the confirmed incidence data (in contrast to Model 3) the LOOCV (Leave-one-out cross-validation) MAE (Mean Absolute Error), 10.93, is inferior to the corresponding model which estimates a culling effect (Model 3), as well as other GLMs such as the original Poisson GLM (Model 1) from [22]
5	generalized Poisson with herd-years-at-risk offset *	−13.7% (−25.2%, −0.4%)	169.7	217.4	the QQ-Plot for simulated residuals indicates no significant over- or underdispersion, no significant outliers, and we fail to reject the KS test for uniformity of the scaled residuals the posterior predictive check indicates a relatively strong degree of correspondence with the observed data LOOCV MAE, LOOCV RMSE (Root Mean Square Error), AICc and BIC are all inferior to the original Poisson GLM (Model 1) from [22]

**Table 1b.** For confirmed herd breakdowns from initial cull until 4 September 2005 within proactive culling areas of the RBCT, we present a range of frequentist models. Various Poisson GLMs were examined across analyses of the RBCT led by the ISG and a final Poisson GLM was fitted to incidence data for the period from initial cull until 4 September 2005 in Donnelly *et al*. [22]. The model was independently audited by a statistical auditor who certified the accuracy of the findings and the associated interpretations [32,33]. In a separate preprint manuscript by Torgerson *et al.* [21], variants of the model were proposed, each of which are denoted by an asterisk (*) below. BIC and AICc denote the Bayesian Information Criterion and small-sample corrected Akaike Information Criterion, respectively. Lower values of each information criterion are better but note that it is not appropriate to compare the information criteria of the normal linear model (which assumes a continuous response and assumes normality of errors in a different model fitting method) with the Poisson-based models.

model	structure	estimated effect of culling (95% CI)	BIC	AICc	diagnostic comments
6	generalized Poisson with herd-years-at-risk offset and without any culling effect *	NA	185.5	184.0	potential model misspecification is identified in the posterior predictive check, as the model-predicted lines do not closely resemble confirmed incidence data (electronic supplementary material, figure S4) the QQ-Plot of simulated residuals, and associated KS test and outlier test, do not indicate any significant over- or underdispersion nor the presence of outliers however, significant quantile deviations are detected in the residual versus predicted plot the LOOCV MAE (13.15) is the worst among all frequentist GLMs considered for the period from initial cull until 4 September 2005
7	generalized Poisson with herd-years-at-risk covariate *	−18.7% (−24.6%, −12.3%)	147.5	217.2	no significant over- or underdispersion, outliers, nor departures from uniformity are detected in the QQ-plot of simulated residuals the residual versus predicted plot indicates a significant result in the quantile test the posterior predictive check displays strong correspondence with the confirmed incidence data the LOOCV MAE (9.95) indicates identical performance to Model 3 (when baseline herds-at-risk was used as a covariate instead of herd-years-at-risk) and superior LOOCV predictive accuracy compared to the model which used herd-years-at-risk as an offset
8	generalized Poisson with herd-years-at-risk covariate without any culling effect *	NA	155.5	154.2	the posterior predictive check implies potential model misfit due to systematic discrepancies between model-predicted data and confirmed incidence no significant over- or underdispersion, outliers or departures from uniformity are detected in the diagnostic checks of simulated residuals
9	normal linear model (incidence per herd year as response) *	−0.5% (−2.5%, 1.6%)	−31.2	−78.9	a basic linear model quantifies a different response of incidence divided by herd years at risk. Thus, CIs have a changed interpretation the underlying assumption is a normal linear relationship between incidence per herd year at-risk and treatment. The modelling assumptions are potentially restrictive and avoidable (in each study period), given the typical appropriateness of negative binomial and other GLM forms for count data (which could potentially be over- or underdispersed) conventional (non-simulated residuals) QQ-plot analysis indicates strong departure from the QQ-line testing the hypothesis of error normality further, we reject the null hypothesis of the Shapiro–Wilk test (*p*‐value = 0.039) for normality of residuals a nonlinear, non-random pattern is present in the residuals versus fitted values plot diagnostic issues are identified for the two Triplet H observations, whose influence is the greatest (based on Cook’s Distance) across observations. The model’s largest absolute residuals (in opposing directions) are attained for these two areas
10	normal linear model (incidence per herd year as response) without any culling effect *	NA	−82.9	−81.4	for the purported *most parsimonious model* discussed in Torgerson *et al*. [21], the model’s leave-one-out accuracy is poor indeed, for the period from initial cull until 4 September 2005, the attained LOOCV measures of RMSE and MAE are the worst/highest attained values (RMSE, MAE) no significant deviation is detected in the QQ-plot of residuals the simulated residual versus predicted plot indicates significant quantiles the triplet F proactive culling observation is identified for having a large relative hat value and hence, is potentially influential

Therefore, as displayed in tables 1a,b, across the proposed GLMs which account for (and estimate) effects of proactive culling, with varying forms of likelihood assumption and varying inclusion of offsets, the estimated effect of proactive culling on incidence is significant and beneficial within proactive culling areas for four of the five GLMs (excluding the statistically inappropriate linear model). More generally, for the period from initial cull until 4 September 2005, no single model performs strongest across the predictive and information criteria of LOOCV RMSE, LOOCV MAE, AICc and BIC. Thus, consistent with any comprehensive investigation of any scientific process, there is no single true model, and we base our understanding and conclusions (see §4) of the incidence data on the consistent, predominant inferences from the several appropriate models which perform well across diagnostics.

In further detail, we note that for the model with the lowest BIC (Model 3), which is the original Poisson GLM used in Donnelly *et al.* [22] but specified in generalized Poisson form, the proactive culling effect on confirmed incidence within proactive culling areas is significant and beneficial, and model diagnostics do not identify model misspecification nor dispersion. Similarly, LOOCV accuracy is relatively strong, compared with other model fits for the study period. We observe that the quasi-Poisson model structure (Model 2) provides substantial flexibility such that the estimated culling effect within proactive culling areas remains beneficial but the standard error associated with the coefficient estimate is inflated and widened such that the estimated culling effect is no longer statistically significant (at a 5% significance level). Nevertheless, diagnostics, such as Cook’s Distance, unveil issues with the influence of individual observations on the flexible quasi-Poisson model structure (electronic supplementary material, figure S1). The influence of single observations is particularly noteworthy given our small dataset which initiates potential for overfitting. Furthermore, the lack of a defined likelihood prevents verification of the distributional assumptions and model suitability, thus potentially limiting validity of applying the model for robust conclusions. In terms of the elementary linear models, and models excluding treatment effects, proposed in a separate preprint manuscript by Torgerson *et al*. [21], there are discernible modelling issues. The issues include unrepresentative posterior predictions, poor LOOCV predictive performance and frequent violations of important model assumptions. The sub-optimal performance of linear models contrasts to the other reported models which retain a clear scientific structure (regarding the treatment) and perform well across diagnostics. Such models enable a representative understanding of the effects of the culling trial on cattle populations within proactive culling areas (see §4).

#### 3.1.2. Post-trial period

We also fitted each frequentist model to confirmed herd breakdowns during the post-trial period, from 1 year after the last cull until 28 March 2013. We present a detailed appraisal of individual models in electronic supplementary material, tables 6*a*,*b*, while sample model diagnostics and checks are visualized in electronic supplementary material, §6.1.2.

The results from the frequentist model fits for the post-trial period reflect a familiar trend of significant, beneficial effects of proactive culling within culling areas. At a 5% significance level, four of the five GLMs (excluding the ill-fitting linear model) with a modelled culling effect yield significant, beneficial estimated effects, similar to the period from initial cull until 4 September 2005 (and from first follow-up cull until 4 September 2005). In further detail, the quasi-Poisson model fit again yields a widened 95% CI (compared to other models for the post-trial period) for the estimated culling effect on incidence within proactive culling areas. The quasi-Poisson model remains excessively sensitive to individual observations of our small dataset, which is a consistent phenomenon across all three studied periods (two during-trial periods and one post-trial period). In terms of model selection, in the post-trial period, some predictive criteria (such as LOOCV accuracy or AICc) no longer indicate superiority of models with treatment effects. It is important to emphasize that any such information criteria are not guaranteed to select the most appropriate model, as these are simply predictive information criteria. Finally, the three lowest BIC values are attained for models which incorporate a culling effect, while the original Poisson GLM from Donnelly *et al*. [22], but specified in generalized Poisson form, again obtains the lowest BIC without any discernible diagnostic flaws (in terms of its model structure and distributional assumptions) across a range of diagnostic checks. We recall here that unlike AIC (and AICc) which measure predictive accuracy, BIC measures goodness-of-fit (specifically the average likelihood of a family).

### 3.2. Bayesian approaches to modelling

#### 3.2.1. From initial cull until 4 September 2005

**Figure 1.**
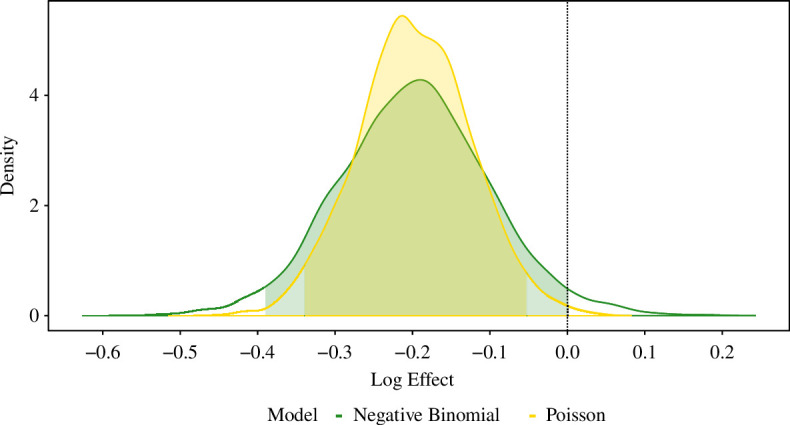
The posterior distribution, alongside 95% CrI, of the treatment effect parameter from the best-fitting Bayesian negative binomial (Model a.2; green) and Poisson (Model e; yellow) GLMs, fitted to confirmed herd breakdowns for the period from initial cull until 4 September 2005. The vertical dashed line located at zero is reflective of hypothetical absence of any culling effect. From the posterior distributions, we deduce that under the models’ conditions, there are 97.5% and 97.6% probabilities, respectively, that the proactive culling effect on incidence was beneficial within proactive culling areas in the period from initial cull until 4 September 2005. We depict the weakly regularizing nature of the prior distribution via a posterior versus prior density plot for the models’ treatment parameters in electronic supplementary material, figure S20, S21.

We fitted Bayesian models to confirmed herd breakdowns from the initial culls until 4 September 2005 (using the same data as presented in Donnelly *et al*. [22]). Tables 2a,b present each model’s key results. In figure 1, we visualize the implied treatment effect within proactive culling areas under the best-fitting Bayesian negative binomial and Poisson GLMs. Sample model diagnostics and checks are displayed in the electronic supplementary material, including visualizations of sensitivity to prior distributions (electronic supplementary material, figures S15–S20) and posterior predictive checks (electronic supplementary material, figures S22, S23).

**Table 2a.** For confirmed herd breakdowns from initial cull until 4 September 2005 within proactive culling areas of the RBCT, we present a range of Bayesian models. The original, frequentist Poisson GLM used in Donnelly *et al*. [22] was re-specified in the Bayesian paradigm in the separate preprint manuscript by Torgerson *et al*. [21], with an alternative negative binomial likelihood (as opposed to Poisson likelihood), and are labelled as Models a.1 and a.2. Models discussed in the separate preprint manuscript by Torgerson *et al.* [21] are denoted by an asterisk (*), and our improved/alternative model versions are denoted by two asterisks (**). With respect to the preprint manuscript by Torgerson *et al*. [21], we employ a different labelling system for models, and the corresponding label from the preprint manuscript can be found in electronic supplementary material, table S2. Estimated culling effects and associated uncertainty are reported using the posterior median and 95% credible intervals (CrIs) of exponentiated posterior samples of the models’ culling effect parameter.

model	estimated effect of culling (95% CrI)	LOO ELPD	diagnostics	conclusions
a.1 (varying intercepts for triplets and covariates of culling effect, historical 3 year incidence of cattle TB, and baseline herds-at-risk) *	−19.1% (−50.9%, 39.3%)	−91.9	the posterior predictive check (electronic supplementary material, figure S13) indicates potential model misfit as the model’s extreme posterior predictions are occasionally too implausibly large for the confirmed incidence data. The implausibility of model-based predictions is an apparent artefact of the strongly informative prior distribution assumption for dispersion which yields extreme overdispersion (electronic supplementary material, figures S17, S18) the posterior predictive intervals are thus extremely wide, and the sample average posterior predictive distribution of our outcome (54.6) is noticeably larger than the mean confirmed incidence (44.1)	under the conditions of the model, there is a 79.9% probability that the proactive culling effect on incidence was beneficial within proactive culling areas
a.2 (a.1 improved) **	−17.9% (−32.3%, 0.0%)	−75.4	the model is improved by specifying weakly regularizing prior distributions (electronic supplementary material, figures S15–S19) including a heavier-tailed, half-Cauchy prior distribution for the auxiliary parameter (reciprocal dispersion) and a scientifically plausible Normal (0,1) prior distribution for the treatment effect the weakly informative nature of the Normal (0,1) prior distribution for the treatment effect (on log scale) is depicted in the prior versus posterior distribution plot (electronic supplementary material, figure S21) consequently, the LOO ELPD value, a measure of the estimated out-of-sample fit and generalizability of the model, improves substantially upon the first negative binomial model structure, and the estimated effective sample sizes for parameters (in particular, the dispersion parameter) also increase furthermore, the sample average posterior predictive distribution (44.4) is far closer to the mean confirmed incidence (44.1) and the posterior predictive check (electronic supplementary material, figure S14) no longer suggests severe model misspecification (implied by previously numerous implausible, extreme incidence values)	the model fit indicates that under the best-fitting negative binomial model (according to estimated out-of-sample generalizability), there is a 97.5% probability that the proactive culling effect on incidence was beneficial within proactive culling areas (figure 1)
b.1 (no varying intercepts for triplets) *	−14.9% (−43.9%, 28.1%)	−81.5	the LOO ELPD value and posterior predictive check indicate superior model fit to confirmed incidence data than the previous unimproved model (Model a.1) for the period from initial cull until 4 September 2005 the posterior predictive intervals are slightly narrowed, and the sample average posterior predictive distribution of the outcome (45.8) is closer to the mean confirmed incidence (44.1) nevertheless, the graphical posterior predictive check hints at model misspecification due to the presence of extreme, highly implausible posterior predictions	under the conditions of the model, there is a 77.9% probability that the proactive culling effect on incidence was beneficial within proactive culling areas

**Table 2b.** For confirmed herd breakdowns from initial cull until 4 September 2005 within proactive culling areas of the RBCT, we present a range of Bayesian models. The original, frequentist Poisson GLM used in Donnelly *et al*. [22] was re-specified in the Bayesian paradigm in the separate preprint manuscript by Torgerson *et al.* [21], with an alternative negative binomial likelihood (as opposed to Poisson likelihood), and are labelled as Models a.1 and a.2. Models discussed in the separate preprint manuscript by Torgerson *et al.* [21] are denoted by an asterisk (*), and our improved/alternative model versions are denoted by two asterisks (**). With respect to the preprint manuscript by Torgerson *et al.* [21], we employ a different labelling system for models, and the corresponding label from the preprint manuscript can be found in electronic supplementary material, table S2. Estimated culling effects and associated uncertainty are reported using the posterior median and 95% CrIs of exponentiated posterior samples of the models’ culling effect parameter.

model	estimated effect of culling (95% CrI)	LOO ELPD	diagnostics	conclusions
b.2 (b.1 improved) **	−13.8% (−31.5%, 7.2%)	−76.1	once again, improving (identically) upon the default prior distribution specifications used in Model b.1, we attain a superior model fit using only weakly informative prior distributions superior model fit is captured by a larger value of LOO ELPD and the posterior predictive check which no longer contains implausible (extreme) synthetic predictions	under the conditions of the model, there is a 91.8% probability that the proactive culling effect on incidence was beneficial within proactive culling areas
c.1 (herd-years-at-risk as an offset and no varying intercepts for triplets) *	−15.0% (−42.2%, 26.4%)	−81.5	the attained LOO ELPD value indicates similar model fit to the previous unimproved models for the period from initial cull until 4 September 2005 the graphical posterior predictive checks hint at model misspecification (electronic supplementary material, figures S22, S23)	under the conditions of the model, there is a 78.4% probability that the proactive culling effect on incidence was beneficial within proactive culling areas
c.2 (c.1 improved) **	−13.6% (−30.6%, 7.7%)	−76.1	once again, improving upon elementary prior distributions such that weakly informative prior distributions are specified, we attain a superior model fit the weakly informative nature of the Normal (0,1) prior distribution for the treatment effect (on log scale) is depicted in the prior versus posterior density plot electronic supplementary material, figure S16 improved model fit is captured by the LOO ELPD and the posterior predictive check, as many seemingly impossible, extreme large posterior predictions are removed (electronic supplementary material, figures S22, S23)	under the model, there is a 91.2% probability that the proactive culling effect on incidence was beneficial within proactive culling areas
d.1 (no culling effect) *	NA	−81.1	the model suffers from apparent severe model misspecification, as the graphical posterior predictive check displays highly extreme and implausible large posterior predictions	under the conditions of the model, we cannot make probability statements about the effect of culling
d.2 - no culling effect (d.1 improved) **	NA	−76.5	introducing the same changes (as before) to our prior distribution specifications, we improve upon the basic model (d.1) in terms of LOO ELPD (and hence, generalizability of the model) however, the graphical posterior predictive check indicates that there is model misspecification as large, extreme posterior predictions remain	from the improved model (where only prior distributions are adjusted as before), we cannot make probability statements about the effect of culling
e (Poisson with baseline herds-at-risk as an offset) **	−13.3% (−24.7%, 0.0%)	−86.5	as an alternative to the negative binomial likelihood, Model e is a fully Bayesian estimation of a Poisson GLM despite the varying likelihood form, the attained LOO ELPD value can be directly compared to the other Bayesian negative binomial GLMs and thus, the Poisson GLM indicates inferior implied out-of-sample predictive accuracy the posterior predictive checks, both in terms of density-based visualizations and distribution of the maximum incidence, indicate that the model recovers plausible posterior predictions of incidence (electronic supplementary material, figures S22 , S23)	under the Bayesian Poisson GLM, there is a 99.5% probability that the proactive culling effect on incidence was beneficial within proactive culling areas (figure 1) the weakly informative nature of the Normal (0,1) prior distribution for the treatment effect (on log scale) is captured by the prior versus posterior density plot (electronic supplementary material, figure S21)

For the period from initial cull until 4 September 2005, across the fitted negative binomial models discussed in the separate preprint manuscript by Torgerson *et al*. [21], we document sub-optimal in-sample predictive performance and apparent model misspecification (via posterior predictive checks). We further note the sub-optimal leave-one-out estimated out-of-sample predictive accuracy, and hence we demonstrate that the generalizability of the models (measured by LOO ELPD) can be readily improved. Our weakly informative (and weakly regularizing) prior distributions in each of our proposed Bayesian models (electronic supplementary material, figures S15–S20) enable superior model fit to within-sample data, reflected by plausible posterior predictive distributions (electronic supplementary material, figures S22, S23). Furthermore, the improved models yield enhanced estimated out-of-sample predictive accuracy, as implied by LOO ELPD values.

In terms of probabilistically quantifying culling effects, under the conditions of any of the improved negative binomial models, there are probabilities of between 91.2% and 97.5% that the proactive culling effect (on incidence) was beneficial in proactive culling areas. The Bayesian Poisson GLM (with an offset for baseline herds-at-risk) produces a higher probability (99.5%) that the proactive culling effect was beneficial, while producing plausible model-based simulated data without systematic discrepancies in the posterior predictive check.

#### 3.2.2. Post-trial period

**Figure 2.**
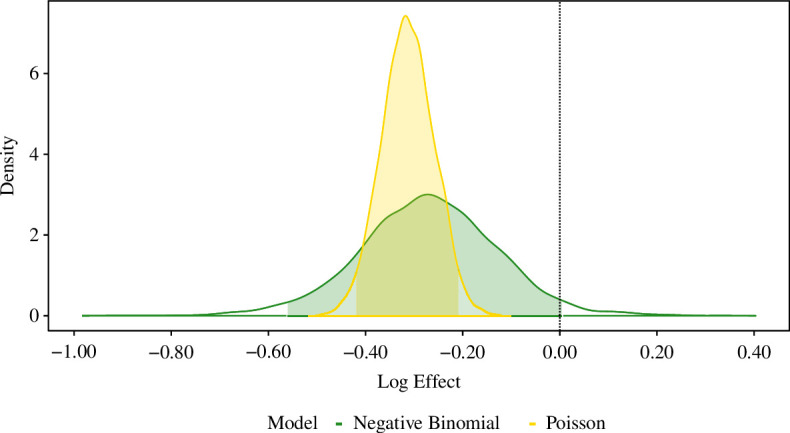
The posterior distribution, alongside 95% CrI, of the treatment effect parameter from the best-fitting Bayesian negative binomial (Model c.2; green) and Poisson (Model e; yellow) GLMs, fitted to confirmed herd breakdowns for the post-trial period (from 1 year after the last proactive cull until 28 March 2013). The vertical dashed line located at zero is reflective of hypothetical absence of any culling effect. From the posterior distributions, we deduce that under the models’ conditions, there are 97.2% and 100% probabilities, respectively, that the proactive culling effect on incidence was beneficial within proactive culling areas.

We also fitted the Bayesian models to confirmed herd breakdowns in the post-trial period; from 1 year after the last proactive cull until 28 March 2013. The same suite of model diagnostics are employed, identical to those performed for the two during-trial study periods. In electronic supplementary material, tables S9a,b, we present the results of each model appraisal. Figure 2 illustrates the implied effects of culling under the best-fitting Bayesian negative binomial and Poisson GLMs.

Leaning on electronic supplementary material, tables S9a,b similar to during-trial studied periods, for the post-trial period, each of the fitted negative binomial models discussed in the preprint manuscript by Torgerson *et al*. [21] suffers from potential model misspecification and sub-optimal estimated out-of-sample predictive accuracy. Our procedure of specifying weakly informative, scientifically plausible prior distributions produces improved model fits, both in-sample and out-of-sample.

Across the improved Bayesian negative binomial GLMs (which have superior estimated model generalizability), conditional on the underlying model assumptions, there are consistently high probabilities of between 97.0% and 99.0% of a beneficial proactive culling effect on confirmed herd breakdowns within culling areas. In particular, the best-fitting Bayesian negative binomial and Poisson GLMs imply probabilities of 97.2% and 100%, respectively, (figure 2) that the post-trial effect of proactive culling on incidence was beneficial within proactive culling areas.

## 4. Discussion

Our extensive analysis has re-evaluated and re-estimated the effects of culling on incidence of herd breakdowns within proactive culling areas, using data across varying study periods, including during- and post-trial time intervals.

For the studied period from initial cull until 4 September 2005 (a period assessed in previous analyses), four of the five frequentist GLMs estimated a significantly beneficial effect of proactive culling on the confirmed incidence of herd breakdowns within culling areas. The exception was the flexible quasi-Poisson model fit (discussed below) which still estimated a beneficial, albeit not significant effect of a 13.6% reduction (95% CI: −31.3%, 8.6%) in confirmed herd breakdowns. Nevertheless, we deduce that across the proposed models, with varying structural forms and assumptions about offsets and covariates, proactive culling was plausibly associated with consistently significant reductions in confirmed herd breakdowns within culling areas. While there existed potential diagnostic issues in the attained residuals of the original Poisson GLM employed in Donnelly *et al*. [22], we similarly observed diagnostic flaws with alternative model formulations. For instance, the greater flexibility enabled by a quasi-Poisson model structure came at the expense of: (i) an inability to validate several important model assumptions (due to no defined likelihood) and (ii) consistently across each studied period, observations (incidence in trial areas) excessively influencing the model fit. The quasi-Poisson model’s flexibility repeatedly yielded discernible statistical flaws for all three studied periods (two during-trial periods and one post-trial period) in terms of excessive influence by individual observations. Thus, in our setting of a small dataset where overfitting is highly conceivable and model selection is generally difficult (due to limited data), we are guided by the consistent evidence implied by our varying frequentist models across the varying time periods.

The inferred consistently beneficial effect of culling on incidence within culling areas was also present in our frequentist analysis of two other studied periods; from first follow-up cull until 4 September 2005 and the post-trial period. For each frequentist Poisson GLM (for which no dispersion parameter is estimated, unless a generalized Poisson form is specified), we reported prudent, overdispersion-adjusted CIs when the estimated overdispersion parameter was greater than 1, as had been performed previously across each of the peer-reviewed analyses of the RBCT data [2,5,18,19,22]. Hence, our re-evaluation of a range of frequentist models, encompassing a diverse range of statistical assumptions and scientific structures, provides an additional layer of robustness to the inferences and conclusions from various aforementioned analyses (of RBCT data) due to the consistency of attained results for the effects of proactive culling within culling areas across three periods (two during-trial and one post-trial).

We expanded our statistical analysis to incorporate a Bayesian perspective for three key reasons: (i) to directly address the uncertainty of inferences (particularly for our small dataset setting), (ii) (relatedly) to make probabilistic statements about the effects of proactive culling, and (iii) to enable comparison of inferences across differing appropriate statistical approaches. Our Bayesian analysis considered the negative binomial models discussed in the preprint manuscript by Torgerson *et al*. [21], while also proposing various model improvements and alternative likelihood forms, as we attempted to ensure the robustness of posterior-based inferences and limit sensitivity to our choice of prior distributions (including likelihoods) and covariates. To validate the statistical assumptions of our proposed models (which only used weakly informative prior distribution specifications), we employed model diagnostics to measure both the fit to observed data and the estimated generalizability of the model, thus enabling us to draw several further, complementary conclusions from our Bayesian approach to analysis.

Irrespective of the Bayesian model rankings implied by information criteria, among the Bayesian negative binomial GLMs with only weakly informative prior distributions, under the conditions of any of these models, there is a probability of between 91.2% and 97.5% that the proactive culling effect (on incidence) was beneficial within proactive culling areas for the period from initial cull until 4 September 2005. The models with weakly regularizing prior distributions improved upon the original Bayesian negative binomial GLMs discussed in the manuscript by Torgerson *et al*. [21] in terms of implied out-of-sample (and in-sample) predictive fit, and are also superior to Bayesian GLMs without a culling effect. The specification of weakly informative and scientifically plausible prior distributions removed important statistical inference issues from models discussed in the preprint manuscript by Torgerson *et al*. [21], including systematic model misspecification (in terms of implausible model-based posterior predictions) and the *a priori* assumption of extreme overdispersion.

We also considered an alternative Bayesian Poisson GLM (again using only weakly informative prior distributions) which produced strong in-sample and out-of-sample predictive fit. For the period from initial cull until 4 September 2005, the Bayesian Poisson GLM (with an offset for baseline herds-at-risk) yielded an even higher probability (99.5%) that the proactive culling effect was beneficial within proactive culling areas. Our conclusions for high probabilities of beneficial effects within culling areas are consistent across models in the period from first follow-up cull until 4 September 2005 and for the post-trial period.

Therefore, we conclude that across a diverse range of substantiated statistical approaches and differing study periods, our attained inferences, of significant beneficial effects on incidence of confirmed herd breakdowns within proactive culling areas, confirm the several peer-reviewed analyses of the RBCT data.

## Data Availability

All code and data used to produce our analysis are available at [34] and [35]. Supplementary material is available online [36].
